# Application of Mechanical Ventilation Weaning Predictors After Elective Cardiac Surgery

**DOI:** 10.5935/1678-9741.20150076

**Published:** 2015

**Authors:** Mayara Gabrielle Barbosa e Silva, Daniel Lago Borges, Marina de Albuquerque Gonçalves Costa, Thiago Eduardo Pereira Baldez, Luan Nascimento da Silva, Rafaella Lima Oliveira, Teresa de Fátima Ramos Ferreira, Renato Adams Matos Albuquerque

**Affiliations:** 1 University Hospital of Universidade Federal do Maranhão (HUUFMA), São Luís, MA, Brazil

**Keywords:** Cardiac Surgical Procedures, Ventilator Weaning, Respiration, Artificial

## Abstract

**OBJECTIVE:**

To test several weaning predictors as determinants of successful extubation after elective cardiac surgery.

**METHODS:**

The study was conducted at a tertiary hospital with 100 adult patients undergoing elective cardiac surgery from September to December 2014. We recorded demographic, clinical and surgical data, plus the following predictive indexes: static compliance (Cstat), tidal volume (Vt), respiratory rate (f), f/ Vt ratio, arterial partial oxygen pressure to fraction of inspired oxygen ratio (PaO_2_/FiO_2_), and the integrative weaning index (IWI). Extubation was considered successful when there was no need for reintubation within 48 hours. Sensitivity (SE), specificity (SP), positive predictive value (PPV), negative predictive value (NPV), positive likelihood ratio (LR+), and negative likelihood ratio (LR-) were used to evaluate each index.

**RESULTS:**

The majority of the patients were male (60%), with mean age of 55.4±14.9 years and low risk of death (62%), according to InsCor. All of the patients were successfully extubated. Tobin Index presented the highest SE (0.99) and LR+ (0.99), followed by IWI (SE=0.98; LR+ =0.98). Other scores, such as SP, NPV and LR-were nullified due to lack of extubation failure.

**CONCLUSION:**

All of the weaning predictors tested in this sample of patients submitted to elective cardiac surgery showed high sensitivity, highlighting f/Vt and IWI.

**Table:** 

**Abbreviations, acronyms & symbols**	
AHCPR	= Agency for Healthcare Policy and Research
CROP	= Compliance, respiratory rate, oxygenation and pressure index
Cstat	= Static compliance
f	= Respiratory rate
f/Vt	= Respiratory frequency to tidal volume ratio
FiO2	= Fraction of inspired oxygen
ICU	= Intensive care unit
IWI	= Integrative weaning index
LR-	= Negative likelihood ratio
LR+	= Positive likelihood ratio
MIP	= Maximal inspiratory pressure
MV	= Mechanical ventilation
NIF	= Negative inspiratory force
NPV	= Negative predictive value
P0.1/MIP	= Occlusion of airway pressure to MIP ratio
PaO_2_	= Arterial oxygen partial pressure
PEEP	= Positive end-expiratory pressure
PPV	= Positive predictive value
PSV	= Pressure support
SaO_2_	= Arterial oxygen saturation
SBT	= Spontaneous breathing trial
Ve	= Minute volume
Vt	= Tidal volume

## INTRODUCTION

Cardiac surgery is a complex procedure that alters several mechanisms required for homeostasis, leading the patient to a critical condition. To ensure adequate recovery, intensive care are needed during post-operative period, including vital signs monitoring and mechanical ventilation (MV)^[1,2]^.

Ventilatory support is often removed right after admission to the intensive care unit (ICU), since the patient is lucid and has hemodynamic stability, receiving low doses of vasoactive drugs^[3-5]^. However, sometimes patients need prolonged MV, which increases both the cost and the risk of complications^[6,7]^.

Ventilator weaning decision must be based not only on clinical judgment^[8,9]^, but also on several predictors that may be applied to support the decision-making process^[10]^. The McMaster Report from the Agency for Healthcare Policy and Research (AHCPR) reviewed and analyzed 66 predictors, but only eight showed consistently significant likelihood ratios: minute volume (Ve); negative inspiratory force (NIF); maximal inspiratory pressure (MIP); airway occlusion pressure at 0.1 second to MIP ratio (P0.1/MIP); static compliance (C_stat_); respiratory rate, oxygenation and pressure index (CROP); respiratory rate (f); tidal volume (Vt); and, in particular, the ratio of respiratory frequency to tidal volume (f/Vt), known as the Tobin Index^[11,12]^.

In 2009, Nemer et al.^[13]^ presented the Integrative Weaning Index (IWI). It evaluates, in a single equation, respiratory mechanics, oxygenation and respiratory pattern through static compliance, arterial oxygen saturation (SaO_2_) and f/Vt [(C_stat_ x SaO_2_)/(f/V_t_)], respectively. Values > 25 ml/cmH_2_O/cycles/min/L may predict weaning success.

Research on MV weaning predictors applied after cardiac surgery are scarce. Therefore, the objective of this study is to test several weaning predictors as determinants of successful extubation after elective cardiac surgery.

## METHODS

This prospective and quantitative study was conducted at a university hospital in São Luís, Maranhão, Brazil. We used a non-probabilistic sample of adult patients submitted to elective cardiac surgery and admitted to the Cardio ICU between September and December 2014. The study was approved by the Institutional Ethics Committee (nº 785.917) and all patients signed an Informed Consent Form.

We excluded patients with neurological, pulmonary or congenital heart diseases and those submitted to emergency surgery. Patients who needed surgical re-intervention, died, required MV over 48 hours after surgery, or had incomplete medical records were also excluded.

Upon ICU admission, all patients received mechanical ventilation performed using Evita 2 dura ventilator (Dräger Medical, Lübeck, Germany) in volume-controlled ventilation mode, with the following parameters: V_t_: 6-8 ml/kg of predicted weight; f: 12 to 16 rpm; PEEP: 8 cmH_2_O; inspiratory flow: 8 to 10 times the minute volume (V_t_ x f); inspiratory time: 1.0 second; and FiO_2_: 40%.

Weaning predictors evaluated and their indicative values of successful extubation are shown in Table 1. Static compliance was obtained directly from MV monitor, thirty minutes after ICU admission.

**Table 1:** Predictive weaning indexes and reference values^[11,12]^

Indexes	Values
f/V_t_	< 105 cycles/min/L
IWI	≥ 25 ml/cmH_2_O/cycles/min/L
Respiratory rate	< 30 rpm
PaO_2_/FiO_2_	> 255 mmHg
Static compliance	> 30 cmH_2_O/L/min
Tidal volume	> 315 ml

f/Vt=Tobin Index; IWI=integrative weaning index; PaO_2_=arterial oxygen partial pressure; FiO_2_=fraction of inspired oxygen

Once the patient began to have spontaneous breaths and presented satisfactory clinical conditions, such as hemodynamic stability, absence or minimal bleeding and adequate level of consciousness (Glasgow Scale > 10), we switched ventilation mode to pressure support (PSV). After 30 minutes with minimal parameters (pressure support: 7 cmH_2_O/Positive end expiratory pressure: 8 cmH_2_O / FiO_2_: 30%), an arterial blood sample was collected to analyze SaO_2_ and PaO_2_/FiO_2_ ratio.

Subsequently, ventilometry was performed to determine minute volume, using an analogical Wright spirometer model Mark 8 (Ferraris Development and Engineering Company Limited, Hertford, England). The patient was instructed to breathe normally for one minute, meanwhile the total amount of exhaled volume and respiratory rate were recorded in order to determine tidal volume (Ve/f) and f/Vt (in liters). Integrative Weaning Index was obtained by the following equation, proposed by Nemer et al.^[13]^: (C_stat_ x SaO_2_)/(f/V_t_).

During the spontaneous breathing test (SBT), the patient was monitored for evidence of weaning failure, such as f > 35 rpm; SaO_2_ < 90%; heart rate > 140 bpm; systolic blood pressure > 180 mmHg or < 90 mmHg; agitation; sweating; and altered level of consciousness^[14]^. If none of these signs were observed and after registering of weaning predictors, patients were extubated. Extubation was considered successful if the patient did not require reintubation within 48 hours^[11]^.

Statistical analysis was performed using Stata/SE 12 (Statacorp, CollegeStation, Texas, USA). Continuous variables are presented as mean and standard deviation, categorical variables as frequencies and percentages. To test normality, we applied Shapiro-Wilk test.

Sensitivity (SE = true positive/true positive + false negative), specificity (SP = true negative/true negative + false positive), positive predictive value (PPV = true positive/true positive + false positive), negative predictive value (NPV = true negative/true negative + false negative), positive likelihood ratio (LR+ = SE/[100-SP]), and negative likelihood ratio (LR- = [100 - SE]/SP) were used to evaluate each index.

## RESULTS

Of the 120 patients initially included, 20 were excluded: 10 due to MV over 48 hours, 5 due to associated congenital heart disease, 3 due to incomplete medical records, and 2 due to death after surgery. Therefore, the final sample was comprised of 100 patients.

Clinical and surgical data are described in Table 2. The sample was predominantly male (60%), with mean age of 55.4±14.9 years. 62% of patients presented low risk of mortality (62%), according to InsCor^[15,16]^. Most common intervention was heart valve surgery (52%). Respiratory variables, as static compliance, airway resistance, minute volume, tidal volume, respiratory rate, oxygen saturation, f/Vt, IWI, and MV duration are shown in Table 3.

**Table 2:** Clinical and surgical data of patients undergoing elective cardiac surgery.

Variables	n (%)	Mean ± SD
Gender		
Male	60 (60)	
Female	40 (40)	
Age (years)		55.4±14.9
BMI (kg/m^2^)		26.3±5.7
InsCor		
Low risk	62 (62)	
Moderate risk	33 (33)	
High risk	5 (5)	
Surgery		
CABG	45 (45)	
Valve repair or replacement	52 (52)	
Aortic surgery	3 (3)	
Drainage tubes		1.8±0.8
Surgery duration (minutes)		252.7±86.7
Pump duration (minutes)		106.9±42.1
Aortic clamp duration (minutes)		79.6±41.4

BMI=Body Mass Index. CABG=coronary artery bypass grafting

**Table 3:** Respiratory data of patients undergoing elective cardiac surgery.

Variables	Mean ± SD
Static compliance (ml/cmH_2_O)	43.3±12.5
Airway resistance (cmH_2_O/L/s)	11.7±10.2
Minute volume (L)	7.5±1.8
Tidal volume (ml)	405.6±97.5
Respiratory rate (rpm)	19.2±4.8
Oxygen saturation (%)	98.3±1.1
MV duration (hours)	18.1±13.6
f/V_t_ (cycles/min/L)	52±21.9
IWI (ml/cmH_2_O/cycles/min/L)	100.3±70.7

MV=mechanical ventilation; f/Vt=Tobin Index; IWI=integrative weaning index

All patients were successfully extubated. Sensitivity, specificity, positive predictive value, negative predictive value, positive likelihood ratio, and negative likelihood ratio are shown in Table 4. All predictors analyzed had high SE and LR+. Other scores, such as SP, NPV and LR- were nullified due to lack of extubation failure.

**Table 4:** Predictive weaning indexes, absolute and percentage values, sensitivity and specificity.

Indexes	Sensitivity (% [IC95%])	Specificity (%)	PPV (%[IC95%])	NPV (%[IC95%])	LR+	LR-
f/V_t_	0.99 [0.94-0.99]	0	1 [0.95-1]	0 [0-0.94]	0.99	0
IWI	0.98 [0.92-0.99]	0	1 [0.95-1]	0 [0-0.80]	0.98	0
Respiratory rate	0.97 [0.90-0.99]	0	1 [0.95-1]	0 [0-0.69]	0.97	0
PaO_2_/FiO_2_	0.97 [0.90-0.99]	0	1 [0.95-1]	0 [0-0.69]	0.97	0
Static compliance	0.86 [0.77-0.91]	0	1 [0.94-1]	0 [0-0.27]	0.86	0
Tidal volume	0.85 [0.76-0.91]	0	1 [0.94-1]	0 [0-0.25]	0.85	0

f/V_t_=Tobin Index; IWI=integrative weaning index; PaO_2_=arterial oxygen partial pressure; FiO_2_=fraction of inspired oxygen

## DISCUSSION

In our study, which tested MV weaning predictors after cardiac surgery, all patients were successfully extubated. This was expected since most of the patients had low risk of mortality. It is known that the objective of intra- and post-operative MV is to guarantee adequate pulmonary ventilation until the patient is clinically able to breathe spontaneously. Thus, weaning must be considered as soon as possible^[5]^.

It is important to mention that little research concerning weaning predictors after cardiac surgery has been found in the literature, emphasizing the importance of our study.

All predictors analyzed showed high sensibility. This result is corroborated by other studies that showed better performance of weaning predictors in patients under mechanical ventilation for short periods, as our sample^[10,17-19]^.

Tobin Index (f/V_t_) is considered the most sensitive parameter for predicting weaning success^[10,12,14,20,21]^, supporting our findings. However, research has demonstrated that this index is not as accurate^[22-26]^. This is explained by differences in the studied populations, which lead to variation in pretest probability and, consequently, test referral bias^[27]^.

Different from our findings, a recent study with 72 patients demonstrated that evolution of breathing pattern, assessed by percent change in f/VT during SBT, was better than a single mensuration. A 5% increase in f/V_t_ after 30 minutes of SBT revealed an area under the ROC curve of 0.83, 83% of sensitivity and 78% of specificity^[28]^.

The IWI is a promising new weaning predictor. Nemer et al.^[13]^ found an area under the ROC curve greater than f/V_t_ (0.96 *vs.* 0.85; *P*=0.003) as well as better SE (0.97), SP (0.94), PPV (0.99), NPV (0.14), LR+ (16.05) and LR- (0.03), with highly accurate values, same as Madani et al.^[29]^. In our study, we found similar SE values for the IWI (SE 0.98), although lower than f/ V_t_ (SE 0.99).

On the other hand, Boniatti et al.^[30]^ evaluated a modified IWI, which utilized peripheral oxygen saturation instead of SaO_2_, and concluded that this index, similar to other predictors, does not accurately predict extubation failure.

Some studies showed that PaO_2_/FiO_2_ ratio was not accurate for predicting successful weaning^[13,31]^. A large variation of its values may predict extubation success (<150 to 300 mmHg)^[10,32]^ and this could explain differing results. Another point that must be considered is the possibility of extubation even with lowerthan-recommended values^[33]^.

Concerning respiratory rate, a recent study reported that the best cut-off value generated by the ROC curve is f < 24 rpm. This result suggests that the cut-off values found in literature are excessively high. In this same study, f was considered an efficient predictor of weaning failure (SE 100%; SP 85%; NPV 100%; PPV 60%, LR+ 6.68; LR- 0; and accuracy 88%, *P*<0.0001)^[34]^.

The small sample is a major limitation of our study. In addition, the lack of extubation failure compromised statistical analysis, although this may be justified by the sample characteristics.

## CONCLUSION

All of the weaning predictors tested in this sample of patients submitted to elective cardiac surgery showed high sensitivity, highlighting f/V_t_ and IWI.

**Table:** 

**Authors' roles & responsibilities**	
MGBS	Study design; implementation of projects/experiments; analysis/interpretation of data; manuscript writing or critical review of its content; final approval of the manuscript
DLB	Study design; implementation of projects/experiments; analysis/interpretation of data; statistical analysis; manuscript writing or critical review of its content; final approval of the manuscript
MAGC	Implementation of projects/experiments; final approval of the manuscript
TEPB	Implementation of projects/experiments; final approval of the manuscript
LNS	Implementation of projects/experiments; final approval of the manuscript
RLO	Implementation of projects/experiments; final approval of the manuscript
TFRF	Implementation of projects/experiments; final approval of the manuscript
RAMA	Implementation of projects/experiments; final approval of the manuscript
